# Dental Anatomy, (Continued)

**Published:** 1911-02-15

**Authors:** N. S. Hoff


### DENTAL ANATOMY.

(Continued.)

By N. S. Hoff, D.D.S.

**Faulty Amelification.** From the prophylaxis view-point, defective enamel is a matter of great concern, and the possibility of preventing defective calcification makes it highly important that we know all that is possible as to the period of calcification and conditions which might produce faulty enamel. It has been generally supposed, that as the enamel organ is composed of epithelial cells that various eruptive diseases to which the child is subjected are liable to interfere with normal calcification. We find, however, that when the process of calcification is most active there is no direct tissue cell connection between the enamel organ and the exterior or epithelial tissues which are most likely to be affected by such diseases, and in many cases of faultily developed enamel there is no history of any such diseases. It may be possible that defective calcification occurs because of lack of normal development of the cells of the enamel organ; therefore any effective preventive treatment will have to be applied long before the birth of the child, or during the period when the tooth organ itself is forming. We should then have to go back at least to the twentieth week of foetal development, as the first traces of enamel deposit appears in the deciduous teeth at that time, and to the time of birth for the first deposit of enamel in the first permanent molars. To apply any treatment that would insure a normal enamel organ and prevent possible aberrations in amelification, we should have to apply prophylactic measures rather early in the gestation period. It would require very keen diagnosing to know when therapeutic intervention should be applied on such a basis. It is more than likely that all aberrations in amelification are simple accidents which may result from the many shiftings of the embryonic cells in the various transformations of organizing into mature tissues, and therefore should be classified as mechanical causes. In this way we could readily account for the absence of tooth germs, or the presence of supernumeraries, as well as the more common deformities in tooth calcification. Just what these mechanical processes may be, and why they happen, it would be indeed difficult to state. That they are promoted by inherited cell taint and hereditary causes is not so readily believed as that they are the result of a lack of nutritional function caused by some systemic disease which interferes with the trophic functions. It has been suggested that the so called Hutchinson teeth are caused by the administration of mercury. In another place this subject will receive further consideration.

**The Dentine Organ.** When the embryonic connective tissue cells begin to differentiate to form the several developmental tissues of the tooth germ, a special organ called the dentine papilla appears with a more or less organized structure, having for its function the creation of the dentine of the crown and root, and the tooth pulp. This papilla assumes the general form of the particular tooth crown to be formed. It is developed from the mesodermic layer of cells and gives the cap form to the enamel organ which drops down over it in close contact. Its outer layer of cells is made up of long cylindrical cells called the odontoblasts, which are the active calcification organs which produce all the dentine for the crown and roots of the tooth. From the peripheral ends of the odontoblasts pseudopodic like projections extend into the calcifying area and become inclosed in the dentine as canals through which the dentine receives its pulpal nourishment. Through these canals sensation is also conveyed to the pulp organ. The odontoblasts as such do not become infiltrated with calcific material, they merely recede as calcification progressee, finally to become a part of the pulp organ, with which they have been intimately connected during the calcifying period. While we frequently encounter defective calcification or dentinification it is not so usual as defective amelification. Dentine is not so dense as enamel and is deposited more rapidly. It is also deposited from mesodermic tissues, whose cells are more uniformly developed and less liable to be affected by diseases which would interfere with their function during the formative or their functionating periods, and they are also less liable to mechanical or traumatic injuries. The dentine is the first hard tissue of the tooth to begin formation and it is the last to be completed. When the root is completed the odontoblast may keep on adding some dentine to the the pulpal walls of the canals for a considerable time; and, under certain diseased conditions, the pulp chambers may be almost entirely obliterated with secondary deposits of dentine, although the canals seldom become entirely closed in normal teeth.

**The Tooth Pulp.** The most vital and important remnant of the papilla is the pulp which usually persists throughout the life of the tooth, unless it becomes diseased and is surgically removed. Structurally the pulp resembles embryonic connective tissue. It is largely composed of branched connective tissue cells and fibrils, and a semifluid interfibrillar ground substance; but the fibrils do not join to form connective tissue fibers, they are either collagenous or reticular fibers. The pulp tissue is richly supplied with blood vessels and nerves, furnishing full nutrition and sensation. The arteries divide into branches and spread out fan-shape, ending in capillaries which form a plexus just beneath the odontoblast cells. Some authors claim to have seen lymphatics in the pulp, but their discoveries have not been verified. As the nerve fibers enter the pulp through the apical foramina they are all medulated, but some immediately, and the rest ultimately, loose their sheaths and divide into long and fine vericose fibers, which form into a loose plexus under the odontoblast cells. Terminal branches pass through the odontoblast layer to be lost on its surface or in the dentinal canaliculi. Efforts to demonstrate the presence of terminal nerve fibers in the dentine have not been successful, although an occasional microscopic slide seems to indicate such a final distribution. The primary function of the pulp is of course to produce dentine; but after maturing the crown and root dentinal portions, it continues, at a much slower rate to deposit on the peripheral canal walls additional dentine. When the pulp is chronically irritated by carious encroachment it seeks to protect itself by throwing out a secondary deposit over the affected area. Because of the rapid advance of caries ordinarily this deposit does not have time to develop; but it is often found associated with slowly approaching dissolution of the tooth from any cause.

**The Alveolus.** The alveolar portion of the jaw is made up of a cellular form of bone, which is developed as needed for the support of the roots of the teeth. Like growing bone in other parts of the body it is subjected to many changes, being formed by the osteoblasts of the periosteum, to be torn down by the osteoclasts only to again be reformed as needed for the various changes which take place in the history of the development and eruption of the teeth. It is first seen as a covered crypt containing the tooth sack of the deciduous tooth. As the tooth crown becomes calcified and its root begins to develop, the top of this bony crypt is dissolved away and the tooth emerges, first into the gum tissue, and as it continues to develop it emerges into the mouth fully developed as to its crown portion. As the root afterward develops it carries the constantly growing alveolus up with it until the root is completed. Owing to the rapid development of the alveolus and only a small portion developing as a subperiosteal tissue, it is not dense in structure and is therefor easily fractured. Because of this fact and also of its being intimately associated with the soft tissues about the teeth and mouth it is often involved in dental and oral diseases. Some authors contend that it is the primary seat of infection in the suppurative form of peridentitis. This may occasionally be the case, but in most cases it becomes involved only because of its intimate relations with the soft structure over and about it. The alveolus is richly supplied with blood vessels and nerves from the intermaxillary sources and possesses a high degree of vitality and recuperative power; except, when infection gains access to it and destroys its nutrient supply. The two most destructive diseases are alveolar abscess from infected tooth pulps and suppurative peridentitis. When these have free external drainage they are never a serious menace; but if no external sinus exists interior inflammation or even bone necrosis may ensue. In old age and because of the attacks of destructive disease of the pericementum the alveolus is more rapidly absorbed than any other portions of the jaws. After the teeth are extracted at any age the alveolar process is soon absorbed and after a few years it disappears entirely. This process is also interesting from another prophylactic standpoint. It is essential that its anatomy and physiology be well, understood because of the necessity for causing its absorption and redeposition in changing the alignment and positions of teeth in correcting their malocclusions.

**Eruption of the Deciduous Teeth.** The deciduous teeth are twenty in number; eight incisors, four canines, and eight molars. The average time of emergence from the gums is, for the incisors, from six to ten months after birth, the lower central incisors usually take their places first to be followed soon by the uppers. The laterals next erupt at from ten to twelve months. The first molars appear the fifteenth to the eighteenth month, and the canines from the sixteenth to the twentieth month, and the second molars from the twentieth month, and the second molars from the twentieth to the twenty-fourth month. These teeth begin calcification before birth and are not wholly calcified when they erupt, the roots not being completed in the canines and molars until three years after birth.

According to Dr. Black, in his Operative Dentistry, page 238, the crown of the central incisor is almost entirely calcified at birth and its root at about one and a half years, the lateral at a little later age, the canine still later and its root not being entirely completed until the third year.

Only a cap of the second molar is completed at birth and its roots are not fully formed until about two and one-half years. This data is only approximate, as in some cases calcification will be completed much earlier and in others much later. This statement will have to serve as a basis for observation and therapeutic interference. The entire deciduous set of twenty teeth should be fully calcified and erupted into normal occlusion at three years of age. The deciduous teeth are seldom in malocclusion because they are developed in the formative jaws without having other developing teeth to crowd them out of their normal places. Individual teeth often get out of alignment or occlusion because their cusps are not sufficiently pronounced to make definite articulation mandatory. The eruption of the deciduous set of teeth is almost always accompanied with more or less serious systemic disorders, although the process itself is a normal and entirely a physiological one. The pain incident to the penetration of the gum by the continued outward pressure of the emerging tooth is so severe as to seriously involve the entire sensory nervous organism. It can be greatly mitigated by the incision of the gum tissue over the emerging tooth, or by the application of soothing or narcotic remedies. There can be no serious objection to lancing the gums at the proper time if it is surgically well done, and much suffering can be avoided in this way. Remedial applications are much slower and they should also be skillfully applied. While the deciduous teeth are usually in normal relations, they are liable to caries, which may result in their complete loss before the permanent teeth are ready to take their places.

**Absorption of Deciduous Teeth.** The deciduous teeth are supplanted by the incoming permanent teeth which approach their places in the arch either directly beneath the deciduous tooth or close beside it. The deciduous root is gradually absorbed by a special organ lying in contact with the apical ends of its roots. The process is never pathologic, as in caries or necrosis, but the entire root is dissolved away with no residue of any of the tooth tissue. The deciduous central incisor is first attacked at about four years of age and is completely absorbed to its crown at seven, when the crown is easily extracted and the permanent tooth is waiting to take its place. Absorption of the lateral begins at five and ends at eight years. The first molar begins to be absorbed at about seven years and is completed at ten years. The canine begins to absorb at nine and is completed at twelve years. Like the development of these teeth this is a fair statement of average periods, but allowance must be made for precocious as well as delayed absorptions.

The maintenance of the deciduous teeth intact and in position and free from destructive influences for their full time is exceedingly important, as this insures proper jaw development for the emergence of the permanent teeth into right articular positions and normal alignment. Caries of the proximal surfaces prevents interstitial bone growth in the jaw, because the normal stimulus of the crowded arches is lacking. Premature loss of the deciduous teeth or serious disease of the soft structures necessarily involved will also stop bone growth, and the spaces left where a tooth has been extracted will shrink upon itself so that there is no room for the incoming permanent tooth. A serious necrosis of the peridental structures of a deciduous tooth may be the means of stopping amelification of the permanent tooth and the results when it erupts is a defective crown.

**Eruption of the Permanent Teeth.** The permanent denture consists of thirty-two teeth; eight incisors, four canines, eight bicuspids and twelve molars. The incisors, canines and bicuspids replace the twenty deciduous teeth, and all the molars develop in the arch distally to the last deciduous molar. The first molar is the first permanent tooth to erupt, which it does at six years. Its germ is formed very early in foetal life but it lies dormant until birth before calcification begins. The second molar erupts at twelve years, and the third molar from sixteen to twenty years, and in some cases long after this. The calcification of the permanent teeth proceed usually in a regular order, but because of the fact that the anterior teeth have to replace the deciduous set, their eruption is not constant or regular. Often the absorption of the deciduous roots will be delayed, consequently there is no space into which the succeeding tooth can erupt, or if it is possible it may emerge either bucally or lingually to the normal and produce malocclusion of the entire denture. The crowns of the teeth calcify in the following order. The central incisor and first molar begin to calcify shortly after birth and the crowns are completed at five years, and their roots at from nine to ten years. The lateral incisors begin to calcify one year after birth and the crowns are completed at six years, and the roots at ten or eleven; they erupt at eight years. The crowns of the canines begin to calcify two years after birth, and the crowns are completed at seven or eight years and the roots at eleven or twelve years. The bicuspids begin to calcify at five years and their crowns are completed at seven or eight and their roots at about twelve years. The second molars begin to calcify at six years, their crowns are completely formed at nine years and their roots at thirteen; they erupt at from twelve to thirteen years. The third molars begin to calcify at nine years and their crowns are completed at eleven years and their roots at sixteen years. Their eruption is very irregular and varies from sixteen years to twenty-five, or much later in some case.

Occasionally there is a complete absence of one or more teeth, most frequently it is the upper lateral incisors, next in frequency it is the third molars. The cause of this phenomena is wholly accidental, and is brought about by some mechanical interuption of the normal developmental processes. Because it is often traced through several generations it has many times been considered as an hereditary principle, but the absence of the same teeth in succeeding offsprings from parents so affected is not constant, and we are compelled to believe that it is purely accidental. The same interference which cause faulty calcification could of course interrupt all calcification by completely destroying the tooth germ. Just how this occurs of course, it will be difficult to establish. Failure to develop a nutrient system when the germ cells are formed may stop the germ development and so the tooth be lost. Sometimes a tooth will be fully calcified and never erupt because of loosing its place and direction in a crowded jaw. The canines and third molars are most frequently delayed in this way. The lateral incisors are also often lost and this fact may be due to their close proximity to the intermaxillary fissures.

